# The Economic and Public Health Burden of Foodborne Illness in Somalia: Prevalence, Costs, and Policy Imperatives

**DOI:** 10.1002/puh2.70097

**Published:** 2025-08-09

**Authors:** Yakub Burhan Abdullahi, Mohamed Mustaf Ahmed, Yusuf Hared Abdi, Sharmake Gaiye Bashir, Naima Ibrahim Ahmed, Mohamed Sharif Abdi

**Affiliations:** ^1^ Faculty of Health Sciences and Tropical Medicine Somali National University Mogadishu Somalia; ^2^ Faculty of Medicine and Health Sciences SIMAD University Mogadishu Somalia

**Keywords:** economic burden, food safety, foodborne illness, low‐ and middle‐income countries (LMICs), public health, Somalia, trade and agriculture

## Abstract

Foodborne illnesses exert a substantial toll on public health and economic systems globally, with low‐ and middle‐income countries (LMICs) like Somalia being disproportionately affected due to fragile health infrastructure, limited regulatory oversight, and high prevalence of informal food markets. This study assessed the prevalence and economic burden of foodborne diseases in Somalia by integrating regional epidemiological data and cost estimates to compensate for national data gaps. Cholera remains the most frequently reported illness, but evidence from sub‐Saharan Africa suggests a broader burden of pathogens such as *Salmonella*, *Escherichia coli*, and *Campylobacter*, whose surveillance remains underdeveloped in Somalia. Direct healthcare costs are substantial, ranging from outpatient care to the treatment of severe complications, such as Guillain–Barré syndrome and hemolytic uremic syndrome. Indirect costs include lost productivity, educational disruption, long‐term disability, and exacerbated poverty cycles. The impact on the livestock‐driven economy is also profound, with repeated export rejections and trade losses highlighting the vulnerability of Somalia's food system. This perspective underscores the urgent need for enhanced surveillance systems, regulatory harmonization, and investment in food safety infrastructure to reduce the disease burden and protect economic stability.

## 1 Introduction

Foodborne illnesses represent a substantial public health challenge globally, disproportionately affecting low‐ and middle‐income countries (LMICs) like Somalia, where they contribute to significant morbidity and mortality [1, 2]. Estimating the economic impact of foodborne diseases is crucial for informed policy‐making, evidence‐based resource allocation, and the development of effective intervention strategies that can address both immediate health needs and long‐term economic sustainability [1].

Somalia faces a particularly complex array of factors that contribute to an elevated burden of foodborne diseases. The country experiences recurrent outbreaks of cholera, with more than 78,000 suspected cases and 1159 deaths reported in 2017, and continued transmission in subsequent years [3, 4]. These outbreaks occur within the context of an underperforming healthcare system weakened by decades of conflict, high rates of malnutrition affecting vulnerable populations, persistent food insecurity, and severely limited access to clean water and adequate sanitation facilities [3, 5, 6]. The 2024 acute watery diarrhea/cholera outbreak, which resulted in at least nine deaths and 474 cases, demonstrates the ongoing nature of this public health challenge [4].

Somalia's food safety regulatory landscape reflects the complexity of governing food systems in a post‐conflict environment with evolving institutional capacities. The food safety control system operates through a multisectoral approach governed by several laws and enforced by various government ministries, departments, and regulatory agencies [2]. The Somali Bureau of Standards, established under the Somali Standards and Quality Control Act No. 27 of 2020, serves as the national food safety authority with core mandates, including protecting consumers from harmful and substandard food products, ensuring compliance with international food safety standards, and conducting risk‐based evaluations and inspections [2, 7]. The Ministry of Livestock, Forestry, and Range operates the Directorate of Animal Health Department, which oversees livestock production enterprises, veterinary extension services, and food hygiene and meat inspection under the Veterinary Law Code 2016 [2]. Disease surveillance and outbreak investigations are coordinated through the Integrated Disease Surveillance and Response (IDSR) system, implemented with WHO support, which enables real‐time information sharing on priority epidemic‐prone diseases through 1969 community health workers deployed across 71 districts and 201 rapid response teams conducting outbreak investigations [8, 9]. However, coordination among these institutions remains insufficient, with weak enforcement of existing legislation and standards, limited human and infrastructure capacity, and inadequate communication between the federal and state government levels [2].

More broadly, in LMIC settings, the challenges contributing to the foodborne disease burden encompass widespread microbial and chemical contamination of food, inadequate food safety systems with limited regulatory oversight, weak surveillance and monitoring capabilities, and poor food handling and storage practices throughout the food chain [2, 10]. Foodborne pathogens may spread through multiple transmission routes, including contaminated water and environmental pathways, and insufficient public awareness and limited food safety education programing further exacerbate disease transmission risks [1]. The informal nature of many food markets in developing countries, including Somalia's significant informal food sector comprising small‐scale vendors, street food sellers, and traditional food markets operating outside formal regulatory frameworks, presents additional challenges in ensuring food safety standards [11].

Addressing these multifaceted challenges requires targeted interventions that consider Somalia's unique epidemiological and socioeconomic circumstances, systematic efforts to fill critical data gaps in disease surveillance and economic impact assessment, and enhanced cooperation among government agencies, international organizations, and private sector stakeholders involved in food safety management [1]. The development and implementation of a comprehensive national food safety policy, as recently undertaken by Somalia in 2024, represents an essential step toward minimizing disease risks, harmonizing interagency efforts, and ensuring public health protection while supporting the country's agricultural trade objectives [2]. Understanding the specific contributing factors and implementing evidence‐based interventions tailored to Somalia's context is fundamental for preventing and mitigating the substantial economic burden of foodborne diseases in this complex humanitarian and development setting [10].

## 2 Challenges in Estimating the Economic Burden

Accurately estimating the economic burden of foodborne illnesses in Somalia faces several key challenges, primarily stemming from limited research and data availability [2]. The National Food Safety Policy highlights a paucity of data and information on the incidence of foodborne disease outbreaks, which impedes thorough economic assessment [2]. This lack of comprehensive data makes it difficult to quantify the direct and indirect costs associated with these illnesses [2]. Underreporting of cases, due to weak surveillance systems, further compounds the problem [1, 2]. Somalia's health information and surveillance system have been weakened by decades of conflict and natural disasters [2, 12]. This results in a significant underestimation of the true number of cases and associated economic impacts [1]. The lack of a comprehensive regulatory framework and insufficient resources further hinder the collection and collation of information on food safety issues [2]. The prevalence of informal food markets presents additional challenges [2, 10]. A significant portion of the population relies on these unregulated markets for their food supply, making it difficult to enforce safety standards and monitor foodborne disease risks [10]. The informal nature of these markets, coupled with weak enforcement of existing legislation, increases the potential for food‐related hazards and further complicates economic assessment [2]. Therefore, the combination of limited data, underreporting, and the dominance of informal markets creates substantial obstacles to accurately assessing the economic burden of foodborne illnesses in Somalia [1, 2, 10].

## 3 Prevalence of Foodborne Illness in Somalia

The burden of foodborne illness in Somalia is most visibly characterized by recurrent cholera outbreaks, which serve as a sentinel for broader systemic vulnerabilities in food and water safety. Between 2017 and 2024, Somalia reported over 78,000 suspected cholera cases, including 1159 deaths, with continued transmission observed in drought‐affected districts and urban centers [3, 5]. The 2024 acute watery diarrhea/cholera outbreak alone resulted in 474 cases and nine deaths, underscoring the persistent threat posed by *Vibrio cholerae* in settings with limited access to safe water and sanitation [6]. Although cholera dominates reported data due to its epidemic potential and high mortality, other enteric pathogens, including *Salmonella* spp., *Escherichia coli*, and *Campylobacter*, likely contribute substantially to Somalia's foodborne disease burden, although their prevalence remains undocumented due to fragmented surveillance systems [3, 6, 13].

Data from neighboring sub‐Saharan African countries provide critical insights into the pathogens likely circulating in Somalia's food supply. In Ethiopia, for instance, *Salmonella Typhi* was detected in 6.5% of food handlers, with intestinal parasites co‐infecting 20.4% of individuals working in catering establishments [14]. Non‐typhoidal *Salmonella* (NTS), a leading cause of invasive bacterial disease in the region, has been reported at median prevalence rates of 44.8 cases per 100,000 person‐years in sub‐Saharan Africa, with case fatality rates exceeding 17% in high‐risk populations, such as malnourished children [13, 15]. Antimicrobial resistance further complicates NTS management, as evidenced by a 68.5% prevalence of multidrug resistance (MDR) in bloodstream isolates across the region [15, 16]. Similarly, enteropathogenic and enteroaggregative *E. coli* strains have been implicated in diarrheal outbreaks linked to contaminated street foods and vegetables in Ghana and Nigeria, with contamination rates exceeding 50% in informal market samples [17, 18]. *Campylobacter* spp., although rarely tested for in Somalia, demonstrate a high prevalence in poultry systems across East Africa, with studies in Kenya identifying the pathogen in 38%–44% of live bird markets, a finding relevant to Somalia's pastoralist communities and urban meat trade [19, 20].

The scarcity of Somalia‐specific data on these pathogens reflects systemic gaps in laboratory capacity and disease reporting, rather than their absence. For example, although Somali surveillance systems prioritize cholera during outbreaks, routine monitoring of bacterial and parasitic foodborne pathogens is limited [10, 13]. This mirrors the challenges observed in other post‐conflict settings, where *Salmonella* and *Campylobacter* infections are often misclassified as “acute watery diarrhea” due to diagnostic constraints [14, 21]. Furthermore, antimicrobial resistance surveillance, critical for managing pathogens such as extended‐spectrum β‐lactamase (ESBL)‐producing *E. coli*, which exhibits a 20.76% prevalence in sub‐Saharan Africa, remains underdeveloped in Somalia [22, 23].

Cholera's prominence in national health reporting thus represents both a direct public health threat and an indicator of broader food safety issues. The same conditions driving cholera transmission—poor hygiene in food preparation, reliance on contaminated groundwater, and unregulated street food vending—create favorable environments for other pathogens [18, 24]. For instance, in the Nile Basin region shared by Somalia, 72.2% of water sources tested positive for *E. coli*, with 22.2% contaminated by *Salmonella* spp., suggesting parallel risks in the Somali water systems [25]. Livestock production, which constitutes 80% of Somalia's agricultural GDP, poses additional risks. Studies in Kenya and Burkina Faso have identified NTS and MDR *Campylobacter* in up to 80% of slaughterhouse samples, which is a concern for Somalia's unregulated meat value chain [19, 25]. Addressing Somalia's foodborne disease burden requires expanding surveillance beyond cholera to include other neglected pathogens. Regional data underscore the urgency: in Ghana, 52% of street foods harbored *Shigella* or *Salmonella*, whereas in Malawi, 21% of diarrheal cases were attributed to *Campylobacter* [16, 17]. Without equivalent monitoring, Somalia remains vulnerable to undetected outbreaks and increasing antimicrobial resistance.

## 4 Direct Costs of Foodborne Illness

The direct economic burden of foodborne illnesses in Somalia manifests through substantial healthcare expenditures, although the full scope of these costs remains underestimated due to systemic underreporting and fragmented health data (Table 1). Medical costs for acute cases typically encompass hospital admissions, diagnostic tests, antimicrobial treatments, and rehydration therapies, with per‐case expenditures in LMICs averaging $36.56 for outpatient care and $159.90 for inpatient management [26]. These figures, derived from regional studies in sub‐Saharan Africa, likely underrepresent Somalia's reality, where limited access to formal healthcare drives many patients to seek costlier private clinics or forgo treatment. For instance, in neighboring Ethiopia, hospitalizations for *Salmonella*‐related gastroenteritis incur median costs of $122 per episode, with diagnostic delays and antimicrobial resistance prolonging illness duration to 7–14 days and escalating expenses [27, 28].

**TABLE 1:** Estimated direct and indirect costs of foodborne illnesses in low‐ and middle‐income countries (LMICs).

Country/Region	Direct costs (USD)		Indirect costs (USD)				
Outpatient care	Inpatient care	Productivity loss	Other indirect		Total annual cost	Cost per case	Source
Multi‐country LMIC average	$36.56	$159.90	Not specified	Not specified	—	$36.56–$159.90	[27]
Ethiopia (national)	$47.79 (iNTS)	$67.00 (specialized hospital)	Included in total	GBS: $2300–$4800	$723 million	$122 (*Salmonella* cases)	[28, 29]
Burkina Faso (national)	Not specified	Not specified	70% of total cost	30% (death risk reduction)	$391 million	Not specified	[28]
Somalia (estimated)	$36.56	$159.90	48% of households spend 10%–15% income on healthcare	Asset liquidation, debt cycles	Not quantified	$110–$4800 (condition‐dependent)	[2]
Sub‐Saharan Africa (regional)	$27.00 (estimated)	$122.00 (median)	$95.2 billion (LMIC total)	School absenteeism, chronic sequelae	—	$44.8 per 100,000 person‐years (NTS)	[2]
All LMICs (WHO/World Bank)	$15 billion annually	—	$95.2 billion annually	Quality of life, trade losses	$110 billion	Not specified	[30, 31]

Abbreviations: GBS, Guillain–Barré syndrome; NTS, non‐typhoidal *Salmonella*.

Post‐infectious complications further amplify direct costs, particularly in populations with high malnutrition rates and compromised immune systems. Approximately 1%–2% of *Campylobacter* infections progress to Guillain–Barré syndrome (GBS), a paralytic disorder requiring intensive neurological care and rehabilitation [32, 33]. Although Somalia lacks GBS‐specific cost data, LMIC studies estimate acute treatment costs at $2300–$4800 per case, with long‐term disability management consuming 15%–30% of annual household incomes [27, 32]. Similarly, hemolytic uremic syndrome (HUS) following enterohemorrhagic *E. coli* (EHEC) infections necessitates dialysis in 40%–60% of pediatric cases, with acute kidney injury (AKI) increasing hospitalization costs by $4411 per episode and mortality risk by fivefold [34, 35]. These sequelae strain Somalia's fragile healthcare infrastructure, where dialysis capacity remains limited to urban centers, and referral systems for specialized care are underdeveloped.

The cumulative financial impact extends beyond the immediate medical bills. Prolonged illness durations, ranging from 5 to 14 days for *Campylobacter* to 3–6 weeks for severe *Salmonella* infections, necessitate repeated clinic visits, displacing limited household resources [28, 35]. In drought‐affected regions, where 48% of families already spend 10%–15% of their income on healthcare, foodborne illnesses exacerbate the cycles of medical debt and asset liquidation [36, 37]. Furthermore, diagnostic limitations in Somalia's laboratories lead to empirical antibiotic use, driving antimicrobial resistance rates that increase treatment failure risks and necessitate costlier second‐line therapies [27, 30]. This complexity underscores the urgent need for integrated cost‐tracking systems to quantify the true burden of foodborne diseases in Somalia's unique epidemiological context.

## 5 Indirect Costs and Socioeconomic Consequences

The socioeconomic repercussions of foodborne illnesses extend far beyond immediate healthcare expenditures, generating substantial indirect costs that perpetuate cycles of poverty and hinder national development. Productivity losses from missed workdays and reduced labor capacity impose a significant burden on Somalia's economy, with the World Bank estimating that unsafe food costs LMICs approximately $110 billion annually in lost productivity and medical expenses [30, 38]. In Somalia, where 48% of households already allocate 10%–15% of their income to healthcare, prolonged illness durations exacerbate financial instability, forcing families to liquidate assets or incur debt to cover basic needs [2, 39]. These losses disproportionately affect agricultural workers, who constitute over 65% of Somalia's workforce, undermining food production and economic output in a sector critical to national GDP [2, 40].

School absenteeism due to foodborne illnesses compounds intergenerational poverty. A 2023 meta‐analysis of sub‐Saharan African studies revealed that children experiencing recurrent diarrheal episodes lose 12–18 school days annually, impairing educational attainment and future earning potential [41]. In Somalia's drought‐affected regions, where malnutrition rates exceed 30%, enteric infections exacerbate nutrient malabsorption, contributing to developmental stunting and reducing adult productivity by 15%–30% [42, 43]. Although school‐based water, sanitation, and hygiene (WASH) interventions in neighboring Mali reduced diarrhea‐related absenteeism by 27%, persistent conflicts and resource limitations in Somalia hinder similar progress, perpetuating cycles of low literacy and economic marginalization [44, 45].

Long‐term health complications amplify these costs, particularly in populations with limited access to specialized care. Approximately 1%–2% of *Campylobacter* infections progress to GBS, a paralytic disorder requiring intensive rehabilitation. In LMICs, acute GBS treatment costs $2300–$4800 per case, with long‐term disability management consuming 20%–35% of annual household incomes [42, 46]. Similarly, HUS following *E. coli* infections leads to chronic kidney disease in 30%–40% of pediatric survivors, necessitating lifelong dialysis unavailable in 90% of Somali healthcare facilities [42, 47]. These sequelae strain familial caregiving networks, often forcing women to abandon income‐generating activities to support ailing relatives, thereby reducing household resilience to economic shocks [39, 44].

The ripple effects of foodborne diseases extend to macroeconomic stability. Repeated enteric infections in early childhood are linked to 8%–12% reductions in adult height and 10%–15% declines in cognitive function, diminishing Somalia's human capital and labor productivity [42, 43]. Chronic aflatoxin exposure, prevalent in maize and groundnuts, contributes to 30%–40% of hepatocellular carcinoma cases in sub‐Saharan Africa, with treatment costs exceeding $1500 per patient, a prohibitive expense in a country where 60% live below the poverty line [42, 48]. Furthermore, food safety scandals erode trade prospects: The 2011 *E. coli* O104:H4 outbreak in Germany, erroneously linked to imported produce, caused $220 million in trade losses for Spanish farmers, a cautionary tale for Somalia's livestock export sector, which generates 80% of foreign currency earnings [28, 42].

Regional data underscore the urgency of addressing these cascading impacts. In Burkina Faso and Ethiopia, foodborne pathogens like NTS and enterotoxigenic *E. coli* reduce national GDP by 0.9%–3.0% annually through healthcare burdens and lost productivity [27]. For Somalia, where 72% of water sources test positive for fecal coliforms, analogous contamination pathways suggest comparable economic losses, further compounded by climate shocks and displacement crises [39, 44]. Mitigating these costs requires integrated policies that prioritize food safety as a cornerstone of public health and economic development, aligning with the World Bank's $13.2 billion investment in African food system resilience [49, 50].

## 6 Burden on the Food Industry and Trade

Somalia's agricultural export sector faces acute vulnerability to food safety‐related trade disruptions, with its livestock industry, contributing 80% of agricultural GDP and 45% of national GDP, particularly exposed to international market fluctuations. This critical economic pillar, which generates 80% of foreign currency earnings through livestock exports to Gulf Cooperation Council (GCC) countries, remains susceptible to export bans and consumer confidence crises. For instance, between 2016 and 2022, 18% of Somali livestock shipments to Saudi Arabia were rejected due to non‐compliance with sanitary standards, costing an estimated $12–$15 million annually [2]. These challenges mirror broader patterns observed in LMICs, where foodborne disease outbreaks and inadequate safety controls have triggered severe economic losses and long‐term market exclusion.

The 1991 cholera epidemic in Peru illustrates the catastrophic trade impacts of foodborne outbreaks in LMICs. Contaminated seafood exports led to $700 million in lost trade revenue, including $32.57 million from frozen marine product exports, whereas tourism revenues halved due to international consumer fears [51, 52]. Similarly, Uganda's fish export sector collapsed in 1998–1999 when the European Union (EU) imposed a hygiene‐related ban, causing annual earnings to plummet from $44 million to zero [53]. The ban persisted for 2 years until Uganda implemented rigorous processing facility upgrades and traceability systems, underscoring the protracted recovery period required to rebuild export markets after safety failures [53, 54]. Ghana's vegetable sector faced analogous setbacks when the EU suspended imports of chili peppers and eggplants in 2015 due to pesticide violations and pest infestations, resulting in $36 million in lost revenue over 2 years [55]. Although the ban was lifted in 2018 after compliance improvements, the episode highlights how recurrent violations erode trade partnerships, a concern for Somalia, where 72% of water sources test positive for fecal coliforms, increasing contamination risks in irrigated crops [2, 56].

**FIGURE 1:**
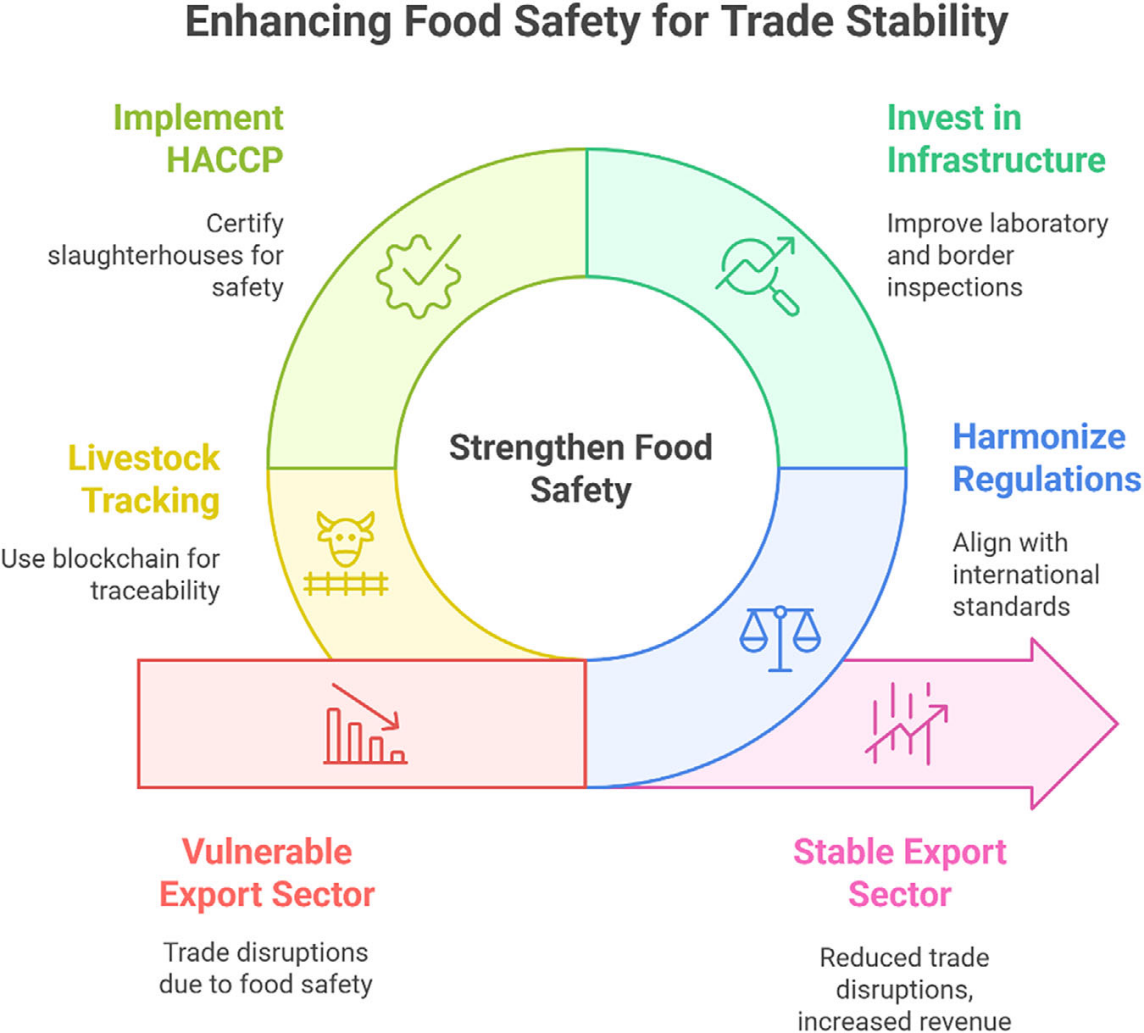
Enhancing food safety for trade stability. HACCP, Hazard Analysis Critical Control Point.

Market access losses often extend beyond immediate revenue declines to include lasting reputational damage (Figure 1). Tanzania's 1997 cholera outbreak, linked to contaminated river water used for irrigation, incurred $36 million in direct trade losses and diminished foreign buyer trust for years [57]. In Cameroon, the EU's 2023 fishery export ban over illegal fishing practices exacerbated existing hygiene‐related restrictions dating to 2004, effectively excluding the country from European markets despite minimal prior trade volumes [12]. These cases demonstrate how food safety incidents compound over time, creating layered barriers to market re‐entry. For Somalia, where informal markets dominate domestic food distribution, the lack of traceability systems heightens risks of undetected contamination cascading into export rejections [2, 58].

The economic repercussions of food safety failures are magnified in LMICs due to their disproportionate reliance on agricultural exports. The World Bank estimates that unsafe food costs LMICs $110 billion annually in lost productivity and medical expenses, with trade‐related losses accounting for 30%–40% of this total [54]. Ghana's 2024 risk assessment projected a potential $50 million annual loss if the EU banned vegetable exports over pesticide misuse, a scenario avoided only through preemptive investments in inspection infrastructure [55]. Such preventive measures remain underdeveloped in Somalia, where overlapping mandates among regulatory agencies and fragmented enforcement undermine compliance with Codex Alimentarius standards [2, 56].

Strengthening food safety systems offers tangible economic returns, as demonstrated by Uganda's post‐ban recovery. After implementing EU‐compliant processing protocols, fish exports rebounded to $149 million by 2001 [53]. Similarly, Ghana's vegetable sector regained $15 million in annual exports post‐2018 by adopting digital traceability tools and farmer training programs [55]. These successes align with Somalia's 2024 National Food Safety Policy, which emphasizes harmonizing regulations with international standards. However, without parallel investments in laboratory capacity and border inspection technologies, Somalia risks repeating the cycles of export bans observed in other LMICs [2, 59]. Proactive measures, including Hazard Analysis Critical Control Point (HACCP) certification for slaughterhouses and blockchain‐based livestock tracking, could mitigate these risks while enhancing competitiveness in Gulf markets [54, 58].

The interplay between food safety and trade stability underscores the necessity of viewing regulatory upgrades as economic investments rather than costs. As global importers increasingly prioritize third‐party certifications and real‐time supply chain monitoring, Somalia's ability to meet these demands will determine its agricultural sector's viability in an era of stringent food safety governance [54, 56].

## 7 Policy Recommendations and Future Directions

### 7.1 Strengthening Regulatory Frameworks and Harmonization

**FIGURE 2:**
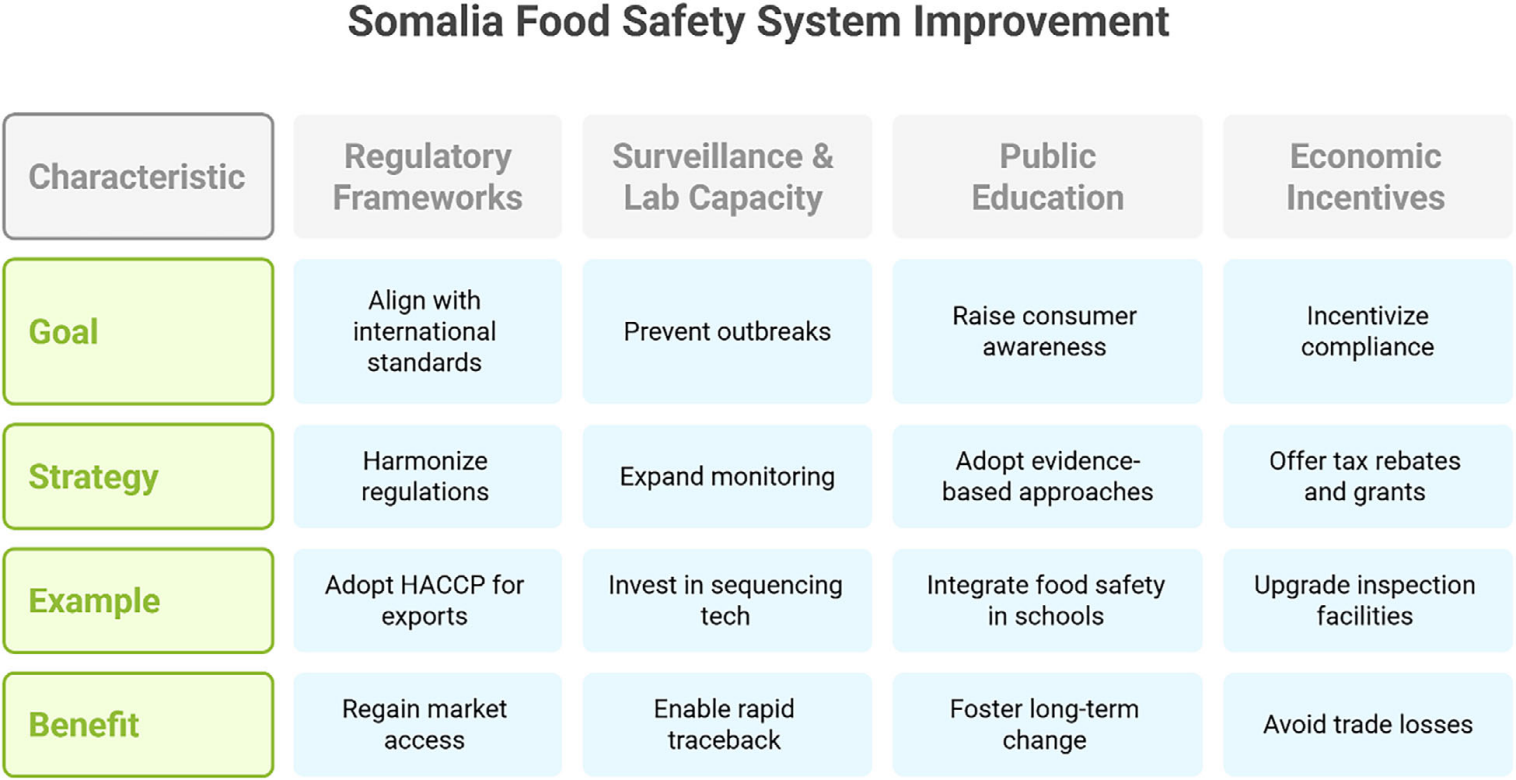
Somalia food safety system improvement.

Somalia's food safety system requires urgent regulatory modernization to align with international standards, such as the Codex Alimentarius, and should be structured through a layered framework of governance, implementation, and community‐level interventions (Figure 2) [60, 61, 62]. The 2024 Somali National Food Safety Policy represents a critical step forward; however, its implementation must prioritize the harmonization of federal and state‐level regulations, as seen in the East African Community's adoption of unified pesticide standards [63]. Learning from Ghana's post‐2018 vegetable export reforms, Somalia should establish a centralized food safety authority with enforcement powers akin to Kenya's Pest Control Products Board, which reduced pesticide violations by 40% through rigorous monitoring [63]. Adopting HACCP certification for livestock exports, as implemented in Uganda's revived fish sector, could help Somalia regain access to Gulf markets while reducing rejection rates [10, 64].

### 7.2 Enhancing Surveillance and Laboratory Capacity

Robust disease surveillance systems are foundational for outbreak prevention, as demonstrated by the United States. The CDC's Integrated Food Safety Centers of Excellence reduced outbreak detection times by 30% through real‐time data sharing [65]. Somalia's IDSR system, supported by the WHO, should expand to include sentinel sites for monitoring pathogens such as *Salmonella* and *E. coli*, mirroring South Africa's National Institute for Communicable Diseases model [10]. Investment in portable sequencing technologies, such as the Whole Genome Sequencing platforms deployed by Africa CDC's One Health Program, would enable rapid traceback of contamination sources [66, 67]. Public–private partnerships could fund regional reference laboratories, following the success of Tanzania's cross‐border aflatoxin testing network, funded by the World Bank [68].

### 7.3 Public Education and Workforce Development

Consumer awareness campaigns targeting high‐risk practices, such as unregulated street food vending, must adopt evidence‐based approaches, such as the WHO Five Keys to Safer Food, which reduced diarrheal incidence by 27% in Mali [61, 69]. Somalia's pastoralist communities would benefit from mobile training modules like Kenya's Micro Enterprises Support Program, which improved smallholder compliance with Good Agricultural Practices through farmer field schools [63]. Integrating food safety into primary school curricula, as piloted in Ghana's Food and Drugs Authority program, could foster long‐term behavioral change while addressing the 52% contamination rate observed in informal markets [10, 70].

### 7.4 Economic Incentives and Trade‐Focused Investments

Financial mechanisms to incentivize compliance should include tax rebates for HACCP‐certified abattoirs and grants for blockchain traceability systems, modeled on Ethiopia's $15 million investment in livestock export infrastructure [64, 68]. The World Bank's $13.2 billion African Food System Resilience Initiative provides a template for leveraging international aid to upgrade Somalia's inspection facilities [69, 71]. Learning from Cameroon's 2023 fishery ban, Somalia must prioritize preemptive investments in export certification systems to avoid losing $12–$15 million annually from Gulf market rejections [64, 68]. The African Union's new Continental Food Safety Agency offers technical assistance to align Somali standards with AfCFTA requirements, which is critical for accessing the $3.4 trillion continental free trade area [64, 71]. The framework emphasizes feedback loops between sectors, mirroring the WHO's “One Health” approach, which reduced zoonotic disease spillover by 22% in Rwanda [66, 67]. Somalia can build a resilient and enforceable food safety system that enables continuous stakeholder engagement, real‐time compliance monitoring, and evidence‐based policy refinement, ultimately enhancing public health and trade competitiveness.

## 8 Conclusion

Foodborne illnesses in Somalia constitute a multifaceted public health and economic crisis, compounded by systemic vulnerabilities, such as under‐resourced health services, weak surveillance, and fragmented regulatory frameworks. Although cholera dominates national reporting, the significant burden of underdiagnosed pathogens and rising antimicrobial resistance remains unaddressed. The direct and indirect economic costs, ranging from high healthcare expenditures and lost productivity to long‐term disabilities and trade disruptions, highlight the far‐reaching consequences of food safety failures. Strengthening food safety governance, investing in diagnostic and surveillance capacity, and engaging stakeholders across the public, private, and community sectors are essential for developing a resilient and health‐protective food system. Prioritizing these actions will not only mitigate the impact of the disease but also enhance trade competitiveness and socioeconomic resilience in Somalia.

## Author Contributions

Yakub Burhan Abdullahi conceptualized and designed the study. Yakub Burhan Abdullahi, Sharmake Gaiye Bashir, Yusuf Hared Abdi, Naima Ibrahim Ahmed, and Mohamed Sharif Abdi conducted literature review and data curation. Yakub Burhan Abdullahi wrote the first draft of the manuscript. Mohamed Mustaf Ahmed critically revised the manuscript for important intellectual content. All authors have read and approved the final manuscript.

## Ethics Statement

The authors have nothing to report.

## Conflicts of Interest

The authors declare no conflicts of interest.

## Date Availability Statement

Not applicable because no new data or databases were used in the preparation of this work.
